# Microglial Sex Differences in the Aging Brain

**DOI:** 10.1093/geroni/igaf122.821

**Published:** 2025-12-31

**Authors:** Seokjo Kang

**Affiliations:** Cedars-Sinai Medical Center

## Abstract

Microglia, the brain’s resident macrophages, are key players in both neuroprotection and neurodegeneration. Microglial heterogeneity is thought to account for at least some of the diverse functional properties of microglia, especially in the context of neurodegeneration. For instance, neuroprotective disease-associated microglia (DAM) become more abundant during healthy aging, as well as in mice and humans with neurodegenerative diseases such as Alzheimer’s disease. Variation in microglial states and subsets may also underlie sex differences in neurodegenerative disease risk. Interestingly, we observed more aging-associated transcriptomic changes in hippocampal microglia from female mice than male mice. We also detected more abundant DAM in the hippocampus of old female mice than old male mice, reflecting a stronger glycolytic shift in females that is promoted by autocrine complement (C3a) signaling to support their phagocytic properties. In ongoing studies, we are evaluating whether these differences are a consequence of more damage in the aging female brain or reveal a stronger neuroprotective response. We are also probing the implications of these findings in the context of healthy aging and aging-associated neurodegenerative diseases.

